# Comparison of rigid and elastic registration methods in software-based targeted prostate biopsy: a multicenter cohort study

**DOI:** 10.55730/1300-0144.5916

**Published:** 2024-09-03

**Authors:** Serhat ÇETİN, Serdar ÇELİK, Murat Yavuz KOPARAL, Güven ASLAN, Sertaç YAZICI, Bahadır ŞAHİN, Sinan SÖZEN, Levent TÜRKERİ

**Affiliations:** 1 Department of Urology, Faculty of Medicine, Gazi University, Ankara, Turkiye; 2 Department of Urology, Faculty of Medicine, University of Health Sciences, Bozyaka Training and Research Hospital, İzmir, Turkiye; 3 Department of Urology, Faculty of Medicine, Dokuz Eylül University, İzmir, Turkiye; 4 Department of Urology, Faculty of Medicine, Hacettepe University, Ankara, Turkiye; 5 Department of Urology, Faculty of Medicine, Marmara University, İstanbul, Turkiye; 6 Department of Urology, Faculty of Medicine, Acibadem University, İstanbul, Turkiye

**Keywords:** Prostate cancer, targeted biopsy, rigid, elastic

## Abstract

**Background/aim:**

This study aims to compare the success rates of rigid registration (RR) and elastic registration (ER) systems in diagnosing all cancers and clinically significant prostate cancer (csPC) in software-based targeted prostate biopsies (TPBs) by performing matching analysis.

**Materials and methods:**

The data of 2061 patients from six centers where software-based TPB is performed were used. All cancer and csPC detection rates of the RR and ER systems were compared following Mahalanobis distance matching with the propensity score caliper method. Logistic regression analysis was applied to identify factors predicting clinically insignificant prostate cancer (ciPC) and csPC diagnoses. Additionally, the International Society of Urological Pathology Grade Group (ISUP GG) upgrade rates of RR and ER systems were compared between biopsy and radical prostatectomy pathologies.

**Results:**

The matched sample included 157 RR and 157 ER patients. No statistically significant difference was found between ER and RR in terms of csPC detection rate (28.0% vs. 22.3% respectively, p = 0.242). The detection rate of all cancers by ER compared to RR was found to be significantly higher (54.8% vs. 35.7% respectively p < 0.001,). No statistically significant difference was found between the ER and RR groups regarding pathological upgrade (39.7% vs. 24.2% respectively, p = 0.130). In the logistic regression analysis performed to determine the factors predicting ciPC, decreased prostate volume and ER system use were found to be independent predictive factors.

**Conclusion:**

While the detection rate of csPC was similar for the RR and ER systems, the detection rate of all cancers and ciPC was significantly higher with the ER systems.

## 1. Introduction

Until recently, transrectal ultrasound-guided 10- or 12-core systematic prostate biopsy (SPB) was the standard approach in patients with elevated prostate-specific antigen (PSA), suspicious digital rectal examination (DRE) findings, or both. The PROMIS study showed that the detection rate of clinically significant prostate cancer (csPC) is lower and the detection rate of clinically insignificant prostate cancer (ciPC) is higher with SPB than with multiparametric magnetic resonance imaging (mpMRI) [1]. Additionally, it was demonstrated by a randomized controlled multicenter study that targeted prostate biopsy (TPB) had a higher success rate of detecting csPC than SPB [2]. In the current European Association of Urology prostate cancer guidelines, prebiopsy mpMRI is strongly recommended for all patients scheduled for prostate biopsy. If lesions with a prostate imaging reporting and data system (PI-RADS) score ≥ 3 are detected, TPB combined with systematic biopsy is strongly recommended for biopsy-naive patients, while TPB alone is weakly recommended for patients with previous negative biopsy [3].

Many commercial platforms exist for software-based TPBs, each with its own specific features. The most important distinguishing features of these platforms are the two techniques of registration of MRI images and real-time ultrasound images, namely rigid registration (RR) and elastic registration (ER). In rigid systems, the prostate borders in MRI images are registered on real-time ultrasound images, regardless of motion or shape changes due to ultrasound probe placement [4]. In elastic systems, the software corrects these shape changes and provides registration [5]. The literature contains cohort and phantom studies comparing these two systems based on csPC detection success, with differing results [6–8].

This study aims to compare the success rates of RR and ER systems in diagnosing ciPC and csPC in software-based TPBs.

## 2. Materials and methods

### 2.1. Study design

This study was created using the Turkish Urooncology Association’s prostate biopsy database. The data of 2061 patients from six centers where software-based TPB is performed were used. Simultaneous SPB was also applied to biopsy-naive patients. No analysis was performed on the SPB results as that is outside the scope of the study. When the data were analyzed retrospectively, it was found that an RR software platform (BioJet, DK Technologies, USA; MIM Symphony DX, MIM, USA; V-Nav, DE Healthcare, UK) was used in three centers; the other three centers used an ER software platform system (UroNav, Invivo, USA in two; Artemis, Eigen, USA in one). The UroNav and Artemis platforms offer both RR and ER methods, so for the three centers where these platforms were used, only patients who underwent biopsy with the ER method were included.

### 2.2. Inclusion and exclusion criteria

Patients having lesions with PI-RADS scores ≥ 3 based on mpMRI and software-based TPB performed between 2016 and 2022 due to elevated PSA levels, suspicious DRE findings, or both were included in the study. Patients with missing data, lesions with PI-RADS scores ≤ 2, or whose registration method was not specified were excluded from the study.

### 2.3. Data collection and definitions

Study data were collected and managed using REDCap electronic data capture tools hosted at the Urologic Cancer Database–Prostate, Urooncology Association, Turkey (UroCaD-P) [9,10]. Clinical data such as age, PSA, and DRE findings; radiological data such as PI-RADS score, prostate volume, index lesion size, index lesion localization (peripheral zone, non-peripheral zone); and pathological data such as the International Society of Urological Pathology grade group (ISUP GG) were obtained from a common database. The mpMRIs of all patients included in the study were reported according to PI-RADS v2.0 or v2.1 [11,12]. Software-based TPB was applied to suspicious lesions detected by mpMRI. In cases of more than one lesion, biopsies were performed by targeting each lesion separately. The International Society of Urological Pathology (ISUP) grading system was used in pathological examinations [13]. In patients with more than one lesion, only index lesion data were included in the analyses. The index lesion was determined by the highest ISUP GG, the highest PI-RADS score, and the largest lesion size. An ISUP GG ≥ 2 is defined as csPC. All cancer and csPC detection rates of the RR and ER platforms were compared. The ISUP GG correlation between the biopsy and prostatectomy pathologies of patients diagnosed with cancer and undergoing radical prostatectomy (RP) was compared in both RR and ER groups. Compared with the biopsy pathology, the increase in ISUP GG in RP pathology was defined as an upgrade. Additionally, logistic regression analysis was applied to identify factors predicting ciPC and csPC diagnoses.

### 2.4. Matching procedure

The matching procedure for this study used the following variables as covariates: age, total PSA, prostate volume, ISUP score, index lesion localization, and index lesion size. The technique used for biopsy (rigid or elastic) was used as the dependent variable in a logistic regression to calculate propensity scores. These scores were then used to determine the nearest Mahalanobis distance between units in the study, and a caliper with a standard deviation of 0.05 was applied to exclude units whose propensity score difference was larger than the caliper. Standardized differences of the covariates were calculated and plotted before and after matching, with any imbalances defined as an absolute value greater than 0.05 being noted.

### 2.5. Statistical analysis

All statistical analyses for this study were conducted using R version 4.0.4 and R Studio version 1.4.1106. The matching analyses were performed using the MatchIt package. Categorical variables were analyzed using the chi-square test and Fisher’s exact test, while continuous variables were analyzed using the Mann–Whitney U test. Simple and multiple logistic regression analyses were performed to identify the parameters predicting the diagnosis of ISUP GG 1 and ISUP GG ≥ 2. The significance level of p = 0.05 was used for all analyses.

### 2.6. Ethics approval

The study protocol was approved by the Clinical Research Ethics Committee of the Gazi University Faculty of Medicine (No: 205 Date: March 06, 2023; Ankara, Turkey)

## 3. Results

### 3.1. Baseline characteristics

Software-based mpMRI-guided TPB was performed on the 2061 patients included in our study. TPB was applied to 1649 (80.0%) patients with software using RR and 412 (19.99%) patients with software using ER. The patient baseline data are summarized in Table 1. As this is a database study, some missing data are indicated in the table.

### 3.2. Matching procedure

Mahalanobis distance matching within the propensity score caliper method was used for matching analysis. RR and ER groups were matched according to age, total PSA, prostate volume, PI-RADS score, index lesion area (apex vs. non-apex), and index lesion size (<10 mm vs. ≥ 10 mm). After matching, 157 RR and 157 ER patients were included in the final sample. Table 2 compares the distributions of the parameters before and after the match. Standardized mean differences were used for a balanced assessment of the match. Figure shows the Love plot of the standardized mean differences. After the match, a sufficient match was shown with standard differences close to zero.

### 3.3. Rigid registration versus elastic registration

No statistically significant difference was found between ER and RR in terms of the csPC detection rate (28.0% vs. 22.3%, p = 0.242, respectively). The rate of detection of all cancers by ER compared to RR was found to be significantly higher (54.8% vs. 35.7%, p < 0.001, respectively). The correlations between biopsy and prostatectomy pathologies of patients diagnosed with cancer and undergoing radical prostatectomy were compared in both groups. No statistically significant difference was found between the ER and RR groups in terms of upgrade (39.7% vs. 24.2%, respectively, p = 0.130; Table 3). In the logistic regression analysis performed to determine the factors predicting ciPC, decreased prostate volume and ER system use were found to be independent predictive factors (Table 4). In the logistic regression analysis for the factors predicting csPC, increasing age, decreasing prostate volume, and increasing PSA density (PSAD) were found to be independent predictive factors, and it was observed that the registration method had no predictive value (Table 5).

## 4. Discussion

This study showed that the RR and ER systems had similar success regarding the csPC detection rate. Additionally, it was concluded that all cancer detection rates were higher with ER systems. Specifically, while the detection rates of csPC were similar, the detection rate of ciPC was found to be higher in ER systems.

Some literature studies compare the success of RR and ER systems. In the first two ex vivo studies, different results were obtained. The first study to compare the success of the two systems was the phantom study published by Westhoff et al., which concluded that the ER system is superior to the RR system regarding targeting the center of the lesion in large prostates with anteriorly located and small lesions [7]. In the phantom study published by Hale et al., while no difference was observed between the two systems in terms of registration error rates, a statistically significantly higher rate of registration error was found in ER systems compared to RR systems in lesions close to the prostate border [6].

A metaanalysis of 21 studies comparing the csPC detection rates of TPB and SPB compared RR results (10 studies) and ER results (11 studies) in different series. This metaanalysis found no difference between the RR and ER systems regarding csPC detection rates; however, both systems were shown to be superior to SPB [14].

While performing real-time ultrasound in RR systems, it is expected that the prostate shape and anatomical localization can change milimetrically due to the placement of the probe in the rectum, resulting in a registration error. The advantage of ER systems is that these changes are detected and corrected by software, promising a more precise registration with real-time ultrasound. Hwang et al., in their study with 19 patients, compared the registration errors of the RR and ER systems using the Euclidean distance measurement method [15]. They compared the RR and ER registry errors with the method they developed without the need for 3D ultrasound [16] and found a significant difference in favor of the ER system (5.32 ± 2.61 mm vs. 2.11 ± 1.37 mm, respectively, p < 0.05) [15].

The literature contains some studies investigating whether the lower misregistration rates provided by the ER system, a product of more advanced technology, reflect meaningfully on clinical practice. A prospective observational study by Sokolokis et al. in 60 patients (40 RR vs. 20 ER) showed that both registration systems had similar cancer detection rates and similarly high ISUP GG detection rates [8]. The same study showed that RR systems have a statistically significant shorter operation time and are more user-friendly, based on evaluation with the System Usability Scale [8,17]. In this study, while csPC detection rates were similar, all cancer detection rates were higher with the ER systems. It was observed that the situation creating the statistical difference is the detection of ISUP GG 1 cancer at a higher rate in ER systems than in RR systems (26.8% vs. 13.4%, respectively, p = 0.003). The logistic regression analysis done in this study showed that decreased prostate volume and ER use are independent predictive factors for ciPC. This situation may be related to clinicians using ER relying on the existence of a more precise registry and sampling close to the lateral margins of the index lesion together with the center. Thus, the detection rate of ciPC in the periphery of the index lesion may have increased due to millimetric registration shifts. However, MRI reporting not being performed by a single radiologist and the associated lack of standardization may have caused this result. Considering that the current approach to prostate cancer supports minimizing detection of ISUP GG 1 cancers, the high rate of ISUP GG 1 by the ER systems in our study can be considered a disadvantage.

The study by Ferriero et al. showed that registration method was not an independent variable in a multivariate logistic regression analysis performed to determine the variables predicting all cancers and csPC after the propensity score match. In the same study, the detection rates of all cancers were 69.8% vs. 56.6% (p = 0.077) for ER and RR, respectively, while the detection rates of csPC were 50.6% vs. 50.6% (p = 1), respectively [18]. That study contains results similar to the findings of this study. Although the difference is not significant, it is observed that the rate of detection of all cancers is higher in the ER system as a percentage. It can also be interpreted that this difference may become significant if the number of patients in the cohort increases. Although ciPC detection rates were not compared in the same study, this rate was found to be higher in the ER group than the RR group in percentage terms (19.2% vs. 6%, respectively) [18]. The retrospective study of Hanske et al. showed that ER system use was associated with a higher rate of detection of all cancers and csPC in the core-based multivariate logistic regression analysis [19].

Another result of our study is the ISUP GG upgrade rates in patients diagnosed with cancer by biopsy and undergoing radical prostatectomy. Although a lower rate of pathological upgrade was detected in patients diagnosed with RR systems compared to patients diagnosed with ER systems, this difference was not statistically significant (24.2% vs. 39.7%, respectively, p = 0.130). In the study by Sussman et al., the pathological upgrade rate in the combined biopsy (TPB + SPB) group was found to be significantly lower than in the group that only underwent SPB (9.89% vs. 22.13%, respectively, p = 0.018) [20]. In the study by Aslan et al., the concordance of 12-core SPB with radical prostatectomy specimens was found to be statistically significantly lower in patients with biopsy pathology ISUP GG 1 compared to combined biopsy [21]. In our literature review, no study was found comparing the ISUP GG concordance between biopsy and radical prostatectomy pathologies of the ER and ER systems.

The most important limitations of this study are that it is retrospective and the biopsies were performed by more than one clinician because of its multicentric nature. Additionally, the interpretation of mpMRIs by different radiologists is a limitation. Moreover, the variability in resolution and Tesla values of MRI devices, differences in the software used for ultrasound-MRI registrations, and the variability in biopsy operators are additional factors contributing to the study’s limitations. However, we believe this situation reflects our daily practice and is valuable in revealing real-life data about the Turkish population. To minimize the effects of the study’s limitations, we aimed to make the heterogeneous structure of the cohort as homogeneous as possible by using the propensity score match method.

## 5. Conclusion

While the detection rate of csPC was similar for the RR and ER systems, the detection rate of all cancers and ciPC was significantly higher for the ER systems. The ISUP GG upgrade rate for patients diagnosed with cancer and undergoing RP is similar for both methods. Randomized controlled studies are required to obtain results with higher levels of evidence.

## Figures and Tables

**Figure:**
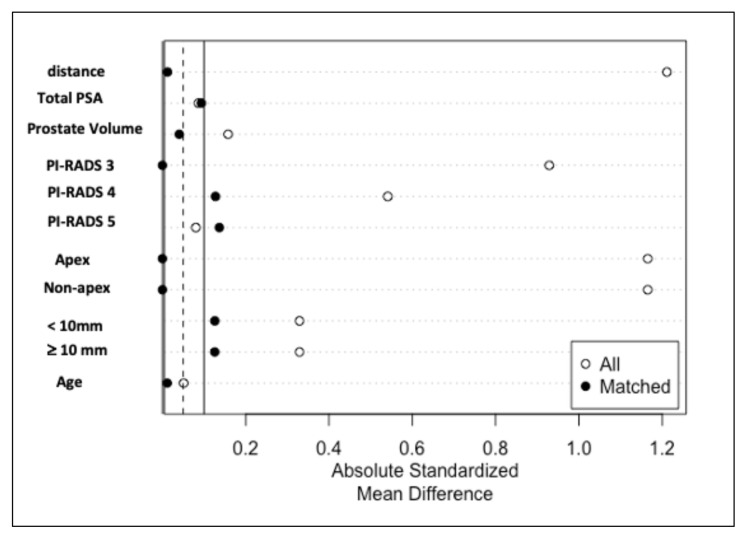
Love plot for absolute standardized mean differences before and after matching.

**Table 1:** Baseline patient characteristics.

	Overall (N = 2061)
**Age**, years, median(IQR)	63(58–68)
**Total PSA**, ng/mL, median(IQR)	6.68(4.87–9.80)
**Prostate volume**, cm^3^, median(IQR)	52.22(44.2–63.11)
**PSAD**, median(IQR)	0.124(0.103–1.151)
**ISUP grade group**, n(%)	
**N-Miss**	2
**0**	1376 (66.8%)
1	282 (13.7%)
2	208 (10.1%)
3	100 (4.9%)
4	43 (2.1%)
5	50 (2.4%)
**PIRADS score**, n(%)	
N-Miss	258
2	31 (1.7%)
3	473 (26.2%)
4	892 (49.5%)
5	407 (22.6%)
**Index lesion localization**, n(%)	
N-Miss	591
Apex	1158 (78.8%)
Nonapex	312 (21.2%)
**Index lesion diameter**, n(%)	
N-Miss	542
<10 mm	478 (31.5%)
≥10mm	1041(68.5%)

**Table 2:** Comparison of unmatched and matched variables.

	Unmatched Group (n = 2061)				Matched Group (n = 314)			
	Rigid (n = 1649)	Elastic (n = 412)	Total (n = 2061)	p value	Rigid (n = 157)	Elastic (n = 157)	Total (n = 314)	p value
**Age, year**				0.297				0.805
Median	63	63	63		64	64	64	
(IQR)	58–68	58–68	58–68		59–69	59–69	59–69	
**Total PSA(ng/mL)**				0.271				0.436
Median	6.68	6.77	6.68		6.90	7.00	7.00	
(IQR)	4.80–9.77	5.00–10.00	4.87–9.80		4.98–9.60	5.28–10.0	5.04–10.0	
**Prostate Volume(cm** **^3^** **)**				**0.006**				0.801
Median	52.2	52.2	52.2		52.2	50.7	52.25	
(IQR)	45.6–62.0	37.3–64.4	44.2–63.1		35.8–72.0	36.4–69.4	35.9–70.4	
**PI-RADS score, n(%)**				**<0.001**				0.511
N-Miss	206	52	258		-	-	-	
2	25 (1.7%)	6 (1.7%)	31 (1.7%)		-	-	-	
3	435 (30.1%)	38 (10.6%)	473 (26.2%)		11 (7.0%)	11 (7.0%)	22 (7.0%)	
4	651 (45.1%)	241 (66.9%)	892 (49.5%)		99 (63.1%)	108 (68.8%)	207 (65.9%)	
5	332 (23.0%)	75 (20.8%)	407 (22.6%)		47 (29.9%)	38 (24.2%)	85 (27.1%)	
**Index lesion localization, n(%)**				**<0.001**				1.000
N-Miss	490	101	591		-	-	-	
Apex	1052 (90.8%)	106 (34.1%)	1158 (78.8%)		85 (54.1%)	85 (54.1%)	170 (54.1%)	
Nonapex	107 (9.2%)	205 (65.9%)	312 (21.2%)		72 (45.9%)	72 (45.9%)	144 (45.9%)	
**Index lesion diameter, n(%)**				**0.004**				0.318
N-Miss	397	145	542		-	-	-	
<10 mm	414 (33.1%)	64 (24.0%)	478 (31.5%)		49 (31.2%)	41 (26.1%)	90 (28.7%)	
≥10 mm	838 (66.9%)	203 (76.0%)	1041 (68.5%)		108 (68.8%)	116 (73.9%)	224 (71.3%)	

**Table 3:** Comparison of cancer detection rates in terms of biopsy and prostatectomy–biopsy correlation for the RR and ER systems.

	Rigid (n = 157)	Elastic (n = 157)	Total (n = 314)	p-value

**csPC**				
**+**	35(22.5%)	44(28%)	79(25.2%)	0.242
**−**	122(77.7%)	113(72%)	235(74.8%)	

**All cancer**				
**+**	56(35.7%)	86(54.8%)	142(45.2%)	**<0.001**
**−**	101(64.3%)	71(45.2%)	172(54.8%)	

**All pathologies**				
**Benign**	101(64.3%)	71(45.2%)	172(54.8%)	**0.001**
**ISUP GG1**	21(13.4%)	42(26.8%)	63(20.1%)	**0.003**
**ISUP GG** ≥ **2**	35(22.3%)	44(28.0%)	79(25.2%)	

**Upgrade**				
**+**	8(24.2%)	25(39.7%)	33(34.4%)	0.130
**−**	25(75.8)	38(60.3%)	63(65.6%)	

**Table 4:** Logistic regression analysis to determine the parameters predicting the diagnosis of ISUP GG 1.

ISUP GG 1 variable	Univariable				Multivariable			
	
	p	OR	95% CIs		p	OR	95% CIs	
	
			Low	High			Low	High

**Age**	0.171	1.028	0.989	1.069				

**PSA**	0.422	0.982	0.933	1.019				

**Volume**	**0.039**	0.895	0.799	0.988	**0.029**	0.889	0.794	0.982

**PSAD**	0.502	1.059	0.885	1.243				

**PI-RADS**								
**3**	ref							
**4**	0.43	1.661	0.534	7.297				
**5**	0.49	1.583	0.468	7.284				

**Rigid/Elastic**	**0.004**	2.365	1.338	4.288	**0.003**	2.419	1.360	4.414

**Index lesion diameter**	0.221	0.692	0.387	1.261				

**Table 5:** Logistic regression analysis to determine the parameters predicting the diagnosis of ISUP GG ≥ 2.

ISUP GG ≥ 2 Variable	Univariable				Multivariable			
	
	p	OR	95% CIs		p	OR	95% CIs	
	
			Low	High			Low	High

**Age**	**<0.001**				**<0.001**	1.031	0.991	1.073

**PSA**	0.06	1.031	1	1.068				

**Volume**	**<0.001**	0.77	0.674	0.865	**0.001**	0.889	0.794	0.982

**PSAD**	**<0.001**	1.477	1.249	1.782	**0.021**	1.233	1.036	1.487

**PI-RADS**								
**3**	ref							
**4**	0.08	5.833	1.170	105.97	0.17	4.099	0.794	75.270
**5**	**0.013**	13.327	2.593	244.51	0.06	6.911	1.272	129.059

**Rigid/Elastic**	0.243	1.357	0.814	2.275				

**Index lesion diameter**	0.448	1.252	0.71	2.273				
